# Solid Lubrication at High-Temperatures—A Review

**DOI:** 10.3390/ma15051695

**Published:** 2022-02-24

**Authors:** Rahul Kumar, Irina Hussainova, Ramin Rahmani, Maksim Antonov

**Affiliations:** 1 Department of Mechanical & Industrial Engineering, Tallinn University of Technology, Ehitajate tee 5, 19086 Tallinn, Estonia; ramin.rahmaniahranjani@taltech.ee (R.R.); maksim.antonov@taltech.ee (M.A.); 2 AC2T Research GmbH, Viktor-Kaplan-Straße 2/C, 2700 Wiener Neustadt, Austria; 3 Laboratory for Nonlinear Mechanics, Faculty of Mechanical Engineering, University of Ljubljana, Askerceva 6, SI-1000 Ljubljana, Slovenia

**Keywords:** self-lubrication, solid lubricants, wear, tribology, high temperature, friction, glaze layer, smart materials

## Abstract

Understanding the complex nature of wear behavior of materials at high-temperature is of fundamental importance for several engineering applications, including metal processing (cutting, forming, forging), internal combustion engines, etc. At high temperatures (up to 1000 °C), the material removal is majorly governed by the changes in surface reactivity and wear mechanisms. The use of lubricants to minimize friction, wear and flash temperature to prevent seizing is a common approach in engine tribology. However, the degradation of conventional liquid-based lubricants at temperatures beyond 300 °C, in addition to its harmful effects on human and environmental health, is deeply concerning. Solid lubricants are a group of compounds exploiting the benefit of wear diminishing mechanisms over a wide range of operating temperatures. The materials incorporated with solid lubricants are herein called ‘self-lubricating’ materials. Moreover, the possibility to omit the use of conventional liquid-based lubricants is perceived. The objective of the present paper is to review the current state-of-the-art in solid-lubricating materials operating under dry wear conditions. By opening with a brief summary of the understanding of solid lubrication at a high temperature, the article initially describes the recent developments in the field. The mechanisms of formation and the nature of tribo-films (or layers) during high-temperature wear are discussed in detail. The trends and ways of further development of the solid-lubricating materials and their future evolutions are identified.

## 1. Introduction

**Figure 1:**
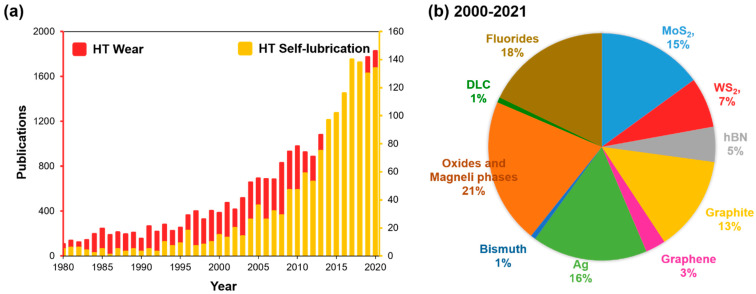
Number of published articles on (**a**) high-temperature wear and self-lubrication (1980–2020); and (**b**) high-temperature self-lubrication based on respective solid lubricants (percentage, 2000–2021), as recovered from Scopus database.

A significant increase in the number of operations performed at high temperatures (HT~upto 1000 °C) has led to an exponential growth of interest in the field of hot tribology (Figure 1a). Wear at HT is a serious concern in a wide variety of technological processes and working systems, including but not limited to material processing, bearings, automotive, metal cutting, hot forging, stamping, forming, etc. In particular, many components function beyond a normal temperature range, unfolding numerous tribological complications, pose a substantial uncertainty in material reliability and performance due to enhanced friction and wear. Changes at tribo-contacts of the interacting bodies and possible new phase formation are common attributes of the HT wear process [1]. The tribo-bodies are highly influenced by a complex transformation of physical, mechanical, and surface reactivity due to simultaneous synergy of oxidation, diffusion, and tribological stress [2]. However, some materials such as steel and its alloys are reported to benefit from the protective nature of the tribo-oxide layer generated over its surface at HT sliding [3]. Nonetheless, easy spallation of the generated tribo-oxide layer owing to its non-adherent nature, ineffective Pilling–Bedworth ratio, or lattice mismatch with a host surface is largely conveyed [4].

Minimizing the wear of tribo-bodies through the application of a conventional liquid lubricant is a common phenomenon. However, the oils and greases limitation are their decomposing at temperatures beyond 300 °C and their harmful effects on environmental and human health [3]. Volatilization, mitigation, and condensation of oil- and grease-based lubricating mediums at extreme conditions (temperature, pressure, altitude) such as in aircrafts, piston–cylinder arrangements, optical or thermal control surfaces, etc. are widely accepted. Considering these limitations of liquid lubricants, if applied in the scenario of extreme working conditions, the durability of the mechanical system as a whole may be limited. The use of solid-based lubricants (SL), such as MoS_2_, WS_2_, graphite, PTFE, Ag, hBN, etc., is a viable solution to minimize friction and wear over a wide range of temperatures from room up to ~1000 °C. Solid lubricants are usually incorporated into the materials (or at the interface of two mating surfaces) in a relative motion, which then is believed to in situ form a lubricious phase or compound due to tribo-chemical reactions at HT [3,4] and to provide a constant transfer of lubricant at the tribo-interface. It is reported that under precise conditions of temperature, humidity, and material composition, they tend to form a ‘glazed’ self-lubricating layer on the material surface under sliding wear [4,5], which offers a significant reduction in coefficient of friction (CoF) and wear. The main advantages of SL over the liquids are better lubricity, good thermal and chemical stability, improved dimensional stability to achieve finishing with high precision, etc.; however, limitations include its inability to carry away heat and provide damping effects during operation [6]. Few works on near-dry or minimum quantity lubrication (MQL) or minimum quantity cooling (MQC) utilizing cutting fluids or vegetable oils in combination with solid lubricants (such as PTFE, hBN, CaF_2_, WS_2_, boron oxide, etc.) during machining of difficult-to-cut materials (Ni superalloy) have come into the picture [6]. However, a lack of promising HT tribological studies in combination with a poor environmental outcome still exists and limits their widespread usage. Figure 1b shows the percentage of research works published concerning notable solid lubricants in HT tribology since 2000–2021.

**Figure 2:**
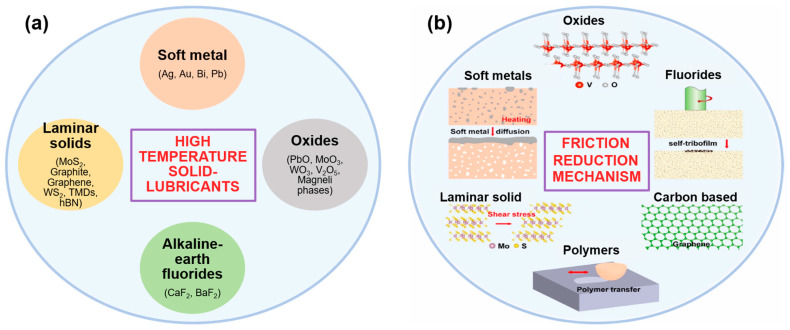
(**a**) Classification of HT solid-lubricants based on their chemical composition; and (**b**) a scheme showing the mechanism of their friction reduction.

In the framework of industrial applications, such as HT forming, forging, stamping, cutting, including areas of relative motion in engines, etc., the foremost importance for a solid-lubricating material is to minimize friction and wear, and also deliver chemical, corrosion, thermal, and mechanical stability. Irrespective of the chosen manufacturing process for material fabrication, low and steady friction in addition to low wear rates must be demonstrated at a wider range of temperatures since the work piece employed during operations can easily extend to or beyond ~1000 °C [7]. This demands the synergetic effect of several solid lubricants in order to achieve a low friction and wear at a large scale of temperatures; as few SLs responsible for minimizing friction at low temperatures are also seen to chemically react and generate a lubricious glazed layer at HT and further enlarge the solid-lubrication range [8]. The key characteristics of an HT solid lubricant (self-lubricating material) are shown in Table 1. Figure 2 represents the classes of HT solid-lubricants based on their chemistry (structure) and their general mechanism of friction reduction, which will be discussed in detail in the subchapters.

This paper is an effort to review the notable SLs (and their based composites) used in tribological applications to impart reduced friction and wear at HT in dry sliding conditions. The mechanism behind their lubricity, chemistry and friction reduction is discussed in detail. A summarized graph showing the range of working temperature for various SLs and their demonstrated CoF during dry sliding is also presented. The idea of a futuristic ‘smart’ tribo-material is introduced.

## 2. Potential High-Temperature Solid-Lubricants

### 2.1. Soft Metals

Soft metals categories an array of materials with relatively low hardness (2.5–4 Mohs), such as gold, silver, lead, bismuth, indium, and platinum. The responsible mechanism of lubrication in soft metals is their greater ductility and low shearing strength [5]. The ease to plastically deform during sliding results in the formation of tribo-surfaces allowing a low coefficient of friction (CoF) and wear. Usually, the dynamic hardness for soft metals is higher as compared to static hardness; therefore, a larger force is required to cause the plastic deformation in a dynamic state [9]. However, an increased softness at HT may result in surface extrusion or failure and, thus, in inefficient lubrication [10]. In general, silver (Ag) and gold (Au) are of great interest in the field of solid lubrication due to a good thermal conductivity in combination with a low shear strength, especially in the areas of a high frictional heat development at the wear interface.

#### 2.1.1. Silver (Ag)

**Figure 3:**
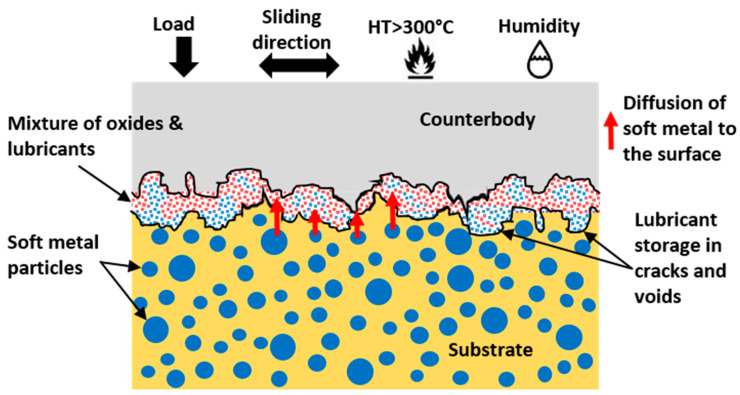
Diffusion mechanism of soft metal-based solid lubrication during HT tribological operations.

Due to its high thermal conductivity (430 W/mK), non-toxicity, and relatively low cost, silver is the most commonly used noble metal as a solid lubricant. However, silver, upon a high inclusion (or coating thicker than 1 µm) in the matrix, can cause high friction and wear rate (in comparison to a virgin substrate). An increased plastic deformation, cutting, plowing, and material transfer to the counter body is commonly reported in such cases [11,12]. Commonly, it is considered that soft metals as reinforcements in matrix tend to be more durable and provide a long-lasting lubricity as compared to the coatings. Quick exhaustion of Ag and its limited lubricity at temperatures above 300 °C, resulting in the coating lapse and increased porosity, is reported in [13]. Figure 3 demonstrates a scheme of lubrication via the diffusion mechanism in a soft metal-based SL.

**Figure 4:**
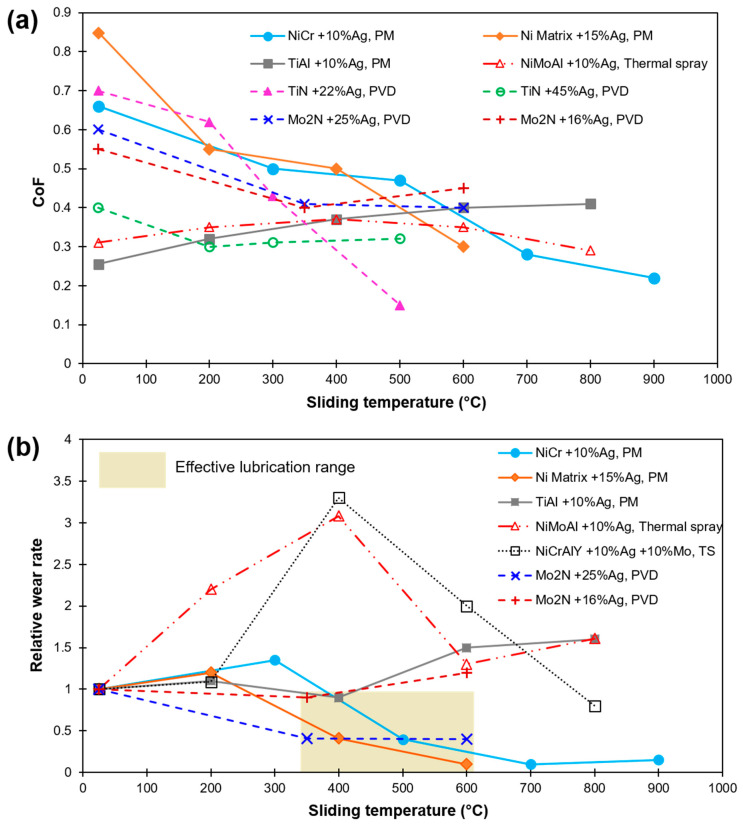
HT sliding results of Ag-based composites and coatings as found in recent literature (**a**) CoF; (**b**) Relative wear rate (in relation to their corresponding RT wear). Values are labeled as per Ag concentration, matrix material, and fabrication method [10,13,14,15,16,17,18,19,20,21].

It is seen that a silver content of ~15 wt% or more in a host material is beneficial in a decrease in CoF and wear with an increase in temperature [14,15]. At temperature < 400 °C, sufficient reduction in CoF and wear of Ag deposited on Al_2_O_3_ substrate is demonstrated [16,17]. However, upon an increase in temperature, the wear rate is accelerated due to expulsion and an increased softening of Ag layers [17]. A reduced and stable CoF at both 400 and 600 °C was reported in Ni-Ag composite [18]. In another study, a significant decrease in CoF and wear was noticed at 200 °C for Ni-Cr alloy-based coating with 10 wt% of each Mo and Ag [19]. However, at a temperature of 400 °C, a high Ag expulsion from the coating results in increased wear. A significant decrease in friction and wear of TiAl alloys incorporated with Ag was demonstrated from room temperature up to 400 °C as compared to a neat alloy in [20]. A five-fold drop in the wear for the Ag-containing films was demonstrated at 600 °C owing to a lubricious tribo-film formation [21]. In most of the works, a 10–15 wt% Ag inclusion was found to be the optimum concentration for wear reduction. Figure 4 shows the CoF and relative wear rates for various Ag-based solid-lubricating materials on a wide range of sliding temperatures, as reported in recent publications [10,13,14,15,16,17,18,19,20,21]. The relative wear rate values are calculated after dividing the wear rate value at the reported temperature by room temperature (RT) value. An efficient lubrication range is shaded in Figure 4b.

A sizeable level of variance in the range of temperature of effective lubrication has been noted. In addition, a disparity in the range of friction and wear values is possible due to the design in the experiment, test methods, and external factors (operator, etc.) in play. Certainly, the microstructure and pre-existing defects (vacancy, lattice mismatch, voids, pores, etc.) can also greatly affect the diffusion of Ag into the surface, resulting in variation in lubricating capacity. However, pores or cracks may also improve the efficiency of lubrication due to the storage of lubricant in the existing defects [22], demonstrating a self-adaptive behavior due to squeezing out or the storing back of the lubricant so as to accommodate the lubricating film for better lubrication (Figure 3).

#### 2.1.2. Bismuth (Bi)

Bismuth has been fairly little recognized and mostly confused with soft metals such as Pb and Ti, which share similar physical properties. In recent years, the combinational use of Bi with Pb/Graphite or Cu as a solid lubricant to improve wear property in the material has come into the picture [23,24]. The restricted use of Pb due to its toxicity has raised the interest in Bi as a ‘green and ecologically clean’ solid lubricant [25].

Bismuth has a low hardness (2–2.5 Mohs) and a low melting point (~270 °C), which results in its easy dispersal under asperity contacts, during which local flash temperatures are high enough for Bi melting [25]. Under tribo-conditions, smearing of generated Bi tribolayer protects the direct contact between tribo-bodies. The bismuth tin bronze with 10% Bi exhibited lower friction in comparison to 5% Bi but underwent shrinkage porosity, and bismuth precipitation on the grain boundaries of the matrix has been specified in [26]. Limited shrinkage porosity was shown by solid-lubricating Cu-Sn bearings produced by powder metallurgy [27]. Bismuth is susceptible to forming grain boundary phases that are unfavorable to the mechanical properties of Cu-Bi alloys. However, Sn is shown to be the best alloying element for preventing Bi precipitation on the grain boundaries [28]. The optimal Bi content for bimetal bronze bearings operating under the boundary lubrication condition is 3 wt% of Bi [29]. The mechanical performance of bismuth bronze alloys, CuSn_10_Bi_4_ and CuSn_6_Bi_6_ in the thrust bearing tests concluded that Bi is not as good dry-lubricant as the lead in the tested alloys due to its poor bearing performance having both low load capacity and a high coefficient of friction (CoF) [30]. Lead (Pb), in addition to Bi and/or graphite, as an inclusion to synergically obtain the best tribo-property, is shown to have the best results.

#### 2.1.3. Other Soft Metals (Au, Cu, In)

The use of electroplated Au-based coating as a solid lubricant in micromechanical and electronics industries is quite common. Gold high ductility and malleability results in easy distribution of frictional stresses during sliding wear conditions [31]. The use of Au in the yttria-stabilized zirconia (YSZ) matrix is shown to improve the sliding wear of YSZ ceramics [32]. Ball-on-disk tribotest results showed that in comparison to reference YSZ ceramic, YSZ/Au coatings demonstrated a significant decrease in CoF and generated less wear debris with limited smearing of Au from the surface. The decrease in CoF (up to 0.2) was owed to the microstructure adaptive changes at elevated temperatures in addition to the formation of lubricious Au transfer films. At least 20 wt% of Au inclusion was stated to cause a diminution of abrasive wear mechanism and impart gold-based lubricity. A considerable decrease in CoF in the range of 0.36–0.5 was recorded from RT-800 °C upon 30 wt.% of CaF_2_ and Au inclusion in a ZrO_2_(Y_2_O_3_) matrix composites [33]. Plastic deformation and material flow were encountered for both CaF_2_ and Au while sliding. Extrusion or transfer of Au from subsurface to the surface resulted in its dispersion and provided enough lubrication during low-temperature sliding. At temperatures of 400 °C or above, the sliding surface showed the existence of both CaF_2_ and Au, pointing to a synergetic solid-lubrication phenomenon. The use of Au in conjunction with other solid lubricants to enlarge the lubrication temperature range has also been reported in [34,35]. However, due to Au’s high cost, its use is limited in large-scale fields.

Copper (Cu) is another member of the soft metal group, which is widely accepted as a solid lubricant. Its high thermal conductivity (398 W/mK) helps to maintain a low temperature at the tribo-pair contact zone. A decrease in CoF in partially stabilized zirconia (PSZ) from 0.40 to 0.20, 0.17, and 0.14 upon copper powder, copper films, and CuO films inclusions/formations, respectively, is reported in [36]. The effect of Cu in brake friction materials has been studied in [37], and the existence of Cu particles within a definite concentration has been stated resulting in the stabilization of sliding by forming a granular layer of the mechanically mixed layer (MML), which are showing main contact sites of pad and disc and further, decreasing the average CoF and fluctuation peaks during sliding. The recrystallized Cu nanoparticles might act as lubricant in the tribolayer formed during sliding at 650 °C [37]. An addition of 40 vol.% graphite to the copper–tin composites showed a low coefficient of friction of 0.15 [38].

Indium (In) based solid lubricants are still scarcely reported. For example, in [39], PVD TiN coatings with indium demonstrated a superior performance up to 450 °C.

### 2.2. Laminar Solids

**Figure 5:**
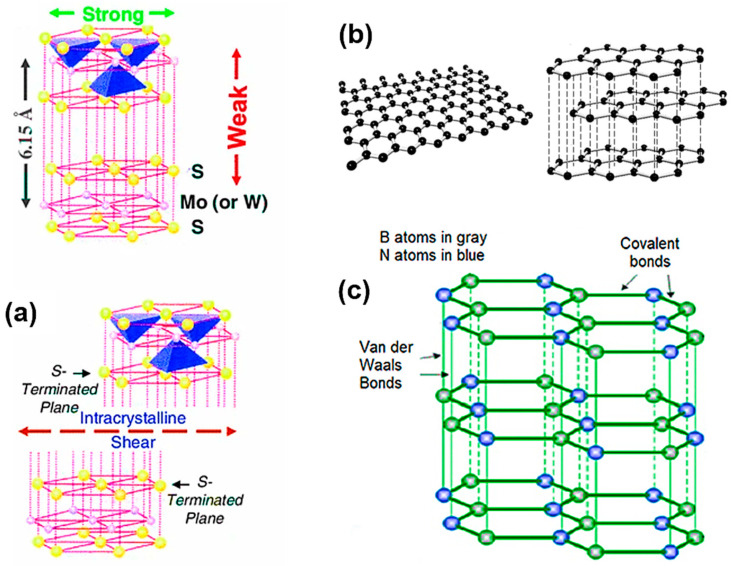
Crystal structures demonstrating the weak Vander walls forces present in the inter-lamellar layers/planers resulting in an easy slip between them (**a**) MoS_2_ (or WS_2_); (**b**) a single layer of graphene and graphite as a heap of multiple graphene layers; and (**c**) hexagonal boron nitride (h-BN) [17].

Laminar solids, also entitled layered lattice compounds, have a planer or hexagonal layered structure, where the atoms within the planes or layers are strongly bonded to each other. However, the bonding between the individual layers or planes is characterized by weaker Vander Wall forces. The weak interlayer forces provide an isotropic shear ability with an easy shear along the basal planes [17]. Hence, this class of materials, when included in the matrix as an SL, offers notable anti-friction capability [3]. Layered sodium silicates such as δ-Na_2_Si_2_O_5_, α-Na_2_Si_2_O_5_, β-Na_2_Si_2_O_5_, and kanemite represent another group of relatively inexpensive materials to reduce wear due to their layered structure similar to other transition-metal dichalcogenides [40]. The widely known materials of this category include graphene, graphite, hexagonal boron nitride (hBN), TMDs, and particularly MoS_2_ and WS_2_ (Figure 5).

#### 2.2.1. Molybdenum Disulfide (MoS_2_)

Until recently, MoS_2_ has been one of the most used solid lubricants worldwide [41]. This compound is found naturally in the earth’s crust as a mineral Molybdenite. Upon refinement and treating, it is commercially accessible in the form of fine particles, suspensions, films, or inclusions in the composites. Considered as a laminar solid, the sulfur lamellae in the compound is bonded by weak van der Waals, which eases the shearing phenomena resulting in layer arrangements (or alignment) during sliding. Moreover, the strong covalent bonding among sulfur and molybdenum provides the lamellae a needed resistance to asperity penetration [41]. The laminar structure is shown in Figure 5a. The mean CoF of unmodified MoS_2_ is about 0.08 at room temperature and up to 300 °C. In a vacuum, MoS_2_ provides acceptable lubrication up to ~1000 °C depending on various factors such as sliding speed, load, and working conditions [42].

The effectiveness of MoS_2_ considerably decreases due to oxidation [3,43]. Formation of molybdenum oxide (MoO_3_) upon oxidation of MoS_2_ results in an increase in friction and wear rate. Abrasive behavior of MoO_3_ to several alloys was reported [41,44]. In contradictory, an improvement in tribological behavior upon MoO_3_ additions to MoS_2_ was seen in ref [45]. Oxidation of MoS_2_ is a function of powder particle size and the accessibility to air [7], as well as the type and composition of inclusion [41]. However, protection from oxidation through the use of inclusions with MoS_2_ is shown [46], resulting in the elimination of air from the particles.

**Figure 6:**
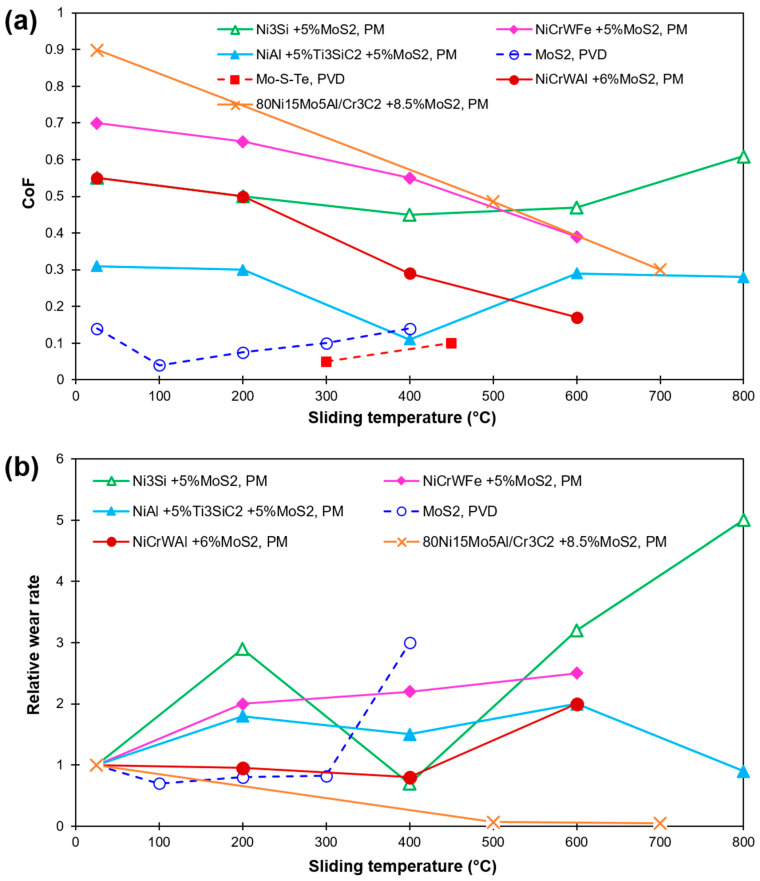
HT sliding results of MoS_2_-based composites and coatings as found in recent literature (**a**) CoF; (**b**) Relative wear rate (in relation to their corresponding RT wear). Values are labeled as per MoS_2_ concentration, matrix material, and fabrication method [47,48,49,50,51,52,53].

NiAl based composites with 5 wt%Ti_3_SiC_2_-5 wt%MoS_2_ demonstrated an outstanding tribo-behavior from RT to 800 °C with a continuous decrease in friction with rising temperature [47]. A noteworthy drop in both CoF and wear rates was noted at 400 °C. Apart from the formation of a self-lubricious layer, the generation of protective oxides of TiO_2_ and SiO_2_ is held to lower CoF and wear. In [48], ZrO_2_/Y_2_O_3_ composites with an inclusion of 10 wt% MoS_2_ and 10 wt% CaF_2_ showed the lowest CoF and wear rate at 200 °C; at higher temperatures up to 1000 °C, oxidation of MoS_2_ to a less lubricious MoO_3_ occurs [49]. A decrease in CoF and wear was accounted for up to 300 °C for YSZ coating with Mo [50]. However, at temperatures beyond 300 °C, a coating failure occurred. In spite of this, MoS_2_ inclusion diminished the CoF up to temperatures of 700 °C, but the initiation of oxides and non-lubricious effect of MoS_2_ was accounted for. In another tribological study [51], Ni_3_Si-based composite coatings with varying content of MoS_2_ and BaF_2_/CaF_2_ showed that the MoS_2_ decomposed into Mo_2_S_3_, resulting in increased friction and wear values (due to the non-lubricant property of Mo_2_S_3_). Further, the composite was seen to demonstrate poor tribological property at HT due to the low content of solid lubricant. However, upon a higher solid lubricant content (i.e., 15 wt.% MoS_2_ and 10 wt.% BaF_2_/CaF_2_), an admirable self-lubricating property was seen as the temperature exceeds 400 °C due to the formation of a glazed layer. A low and stable CoF~0.15 of PVD films with MoS_2_ was noted at 350 °C. However, a degradation of MoS_2_ to MoO_3_ was reported at around 370 °C [52,53]. Figure 6 demonstrates the effect of sliding temperature over CoF and relative wear rates for various MoS_2_ based solid-lubricating materials reported in recent literature [47,48,49,50,51,52,53]. The relative wear rate values are calculated after dividing the wear rate value at the reported temperature by room temperature (RT) value.

#### 2.2.2. Graphite

Graphite is a layered solid and an allotrope of carbon with a hexagonal lattice arrangement (Figure 5b). The carbon atoms in the layers are strongly held by a covalent bond, while the individual layers held themselves by weak van der Waal cohesive forces, ensuing an easy shear. Its high thermal conductivity (470 W/mK) raises its demand for HT applications. Unlike MoS_2_ and WS_2_, the existence of water vapor and oxygen in the environment aids the interlamellar shearing of graphite crystals and demonstrates lubricity [3]. On the other hand, oxidation of graphite at elevated temperatures is the foremost hindrance to its use [54]. As per reported data, graphite undergoes oxidation to CO beyond 400 °C and even CO_2_ at temperatures beyond 500 °C [55]. Due to this, graphite is majorly employed at medium-range temperatures. Nevertheless, its superior mechanism of lubrication has compelled researchers to look for a way to stabilize graphite at higher temperatures [56]. Coating graphite with a ‘protective barrier’ of W, Re, Mo, Nb, Hf, Ti, Zr, their oxides, silicides, borides, carbides, nitrides, and the respective composites, which would rather hinder its contact with atmospheric oxygen and thus, improve oxidation resistance is seen [55,56,57]. Amongst which, SiC [56] and MoSi_2_ [58] are the most efficient at HT oxidation. Admirable lubrication without adsorption of water vapors and oxidation of graphite fluoride (CF_x_)_n_ was earlier reported in [59], whereas the formation of a lubricious glazed layer in the presence of humid air accounted for the decrease in CoF and the wear in Fe-Cu-Sn alloy with 3 wt% graphite content at 423 K [59].

A synergism of graphite and inter-metallic Al_2_Cu was reported to improve friction and wear in comparison to the base alloy, Al-20Si-5Fe-2Ni [58]. Conversely, another synergetic effect of Ag, BaF_2_/CaF_2_ with graphite inclusion in Ni-alloy matrix was reported to increase the CoF of composites with graphite addition at HT up to 500 °C [60]. An excellent diminution in CoF and wear from RT to 600 °C in addition to an improvement in compressive strength and hardness of graphite incorporated composites was reported in [61]. A synergetic influence of three solid lubricants, i.e., graphite, Sb_2_S_3_, and MoS_2_, in the brake friction material to improve friction stability and fade resistance in comparison to material without graphite was reported in [62]. A similar synergetic influence of Mo, CaF_2_, and graphite was reported to provide improved lubrication due to graphite inclusion at a temperature range up to 400 °C due to the formation of CaF_2_ and CaMoO_4_ during a tribo-reaction of Mo and CaF_2_. Severe brittle fracturing and delamination were seen at 400 and 600 °C. However, from 800 to 1000 °C, the worn surface was covered with the lubricating film [63]. An outstanding solid-lubricating behavior over a varied range of temperature up to 600 °C was shown in [64] due to the synergistic lubricating effect of graphite and MoS_2_ on Nickel-based composites prepared using the PM route. The main mechanisms of wear at RT were adhesive and abrasive. However, at elevated temperatures, the mechanisms were suppressed significantly. Several other studies [65,66,67] discuss the synergetic effect of lubrication of graphite and other solid lubricants. However, a good explanation regarding the occurring synergetic wear mechanisms at HT is scarcely reported.

#### 2.2.3. Graphene

Graphene is an allotrope of carbon with a two-dimensional honeycomb structure and is believed to offer outstanding friction-reducing properties. The mechanism of friction reduction during sliding is the same as graphite and MoS_2_. However, unlike graphite, graphene shows lubrication in a dry environment. Due to its exceptional thermal (~4000 W/mK), electrical (~10^2^ S/m), and mechanical properties, it is widely used as a lubricant in mechanical as well as electronics industries. Its use as in solid or colloidal liquid-based lubricant is also well-recognized [68,69]. High strength, easy shear ability, and chemical inertness have made it a perfect choice in the solid lubricant category. In addition, due to its super-thin dimension, it is widely used in micro and nano mechanical systems [70].

Outstanding tribological performance of graphene nanoplatelets (1.5 wt%) reinforced NiAl matrix due to the formation of a lubricious tribo-layer up to 400 °C reduced CoF and the wear significantly [71]. However, with the rise in sliding temperature up to 500 °C, the protective behavior of graphene nanoplatelets (GNPs) diminishes/dies out due to its oxidation, resulting in intensive adhesive wear and delamination. In another study [72], multilayer graphene reinforced TiAl matrix composite (MLG-TiAL) was reported to demonstrate excellent lubrication from 100 to 550 °C due to graphene’s excellent shear ability. Nonetheless, a loss in the lubrication above 600 °C due to its oxidation resulted in unstable friction and intensified wear. In most of the works regarding graphene as a solid lubricant, a transition period of an increase in friction and wear between 550 and 600 °C is noticed. A study on the wear-reducing behavior of graphene layers and its oxide demonstrated that the former delivered the best wear protection, reducing the wear by 3–4 orders of magnitude in comparison to bare steel sliding interfaces. At the same time, graphene oxide demonstrated a larger wear rate by 1–2 orders of magnitude in comparison to that of graphene layers [73]. Very limited research on graphene as a solid lubricant state, its friction diminishing behavior is up to 500 °C, above which is termed to be severe for their composites due to its degradation.

#### 2.2.4. Hexagonal Boron Nitride (hBN)

hBN is a laminar solid reported to demonstrate lubricity due to its easily sheared ‘graphite like’ layered structure [3]. hBN, due to its high thermal conductivity (~500 W/mK at RT), chemical and oxidation resistance is a potential candidate for HT tribological applications [74]. hBN, unlike graphite and MoS_2_, is understood to be very well effective at HT applications (such as for metalworking processes where lubrication at high temperatures is often sought) and does not appreciably oxidize up to 1000 °C [74]. Nevertheless, non-wettability and poor sinterability of hBN accompanied with its poor adhesiveness to most of the metals and ceramics results in low strength and inferior quality composites or coatings hindering its wide-scale usage.

**Figure 7:**
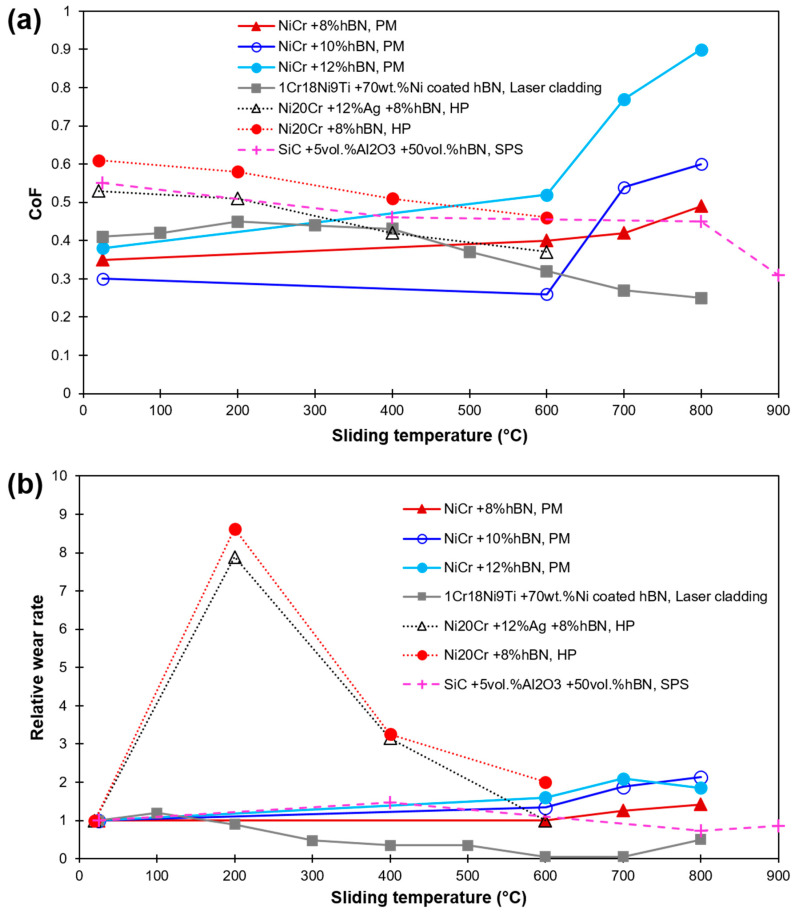
HT sliding results of hBN-based composites and coatings as found in recent literature (**a**) CoF; (**b**) Relative wear rate (in relation to their corresponding RT wear). Values are labeled as per hBN concentration, matrix material, and fabrication method [80,81,82,83,84,85,86].

So far, the results on tribological studies of hBN as a solid lubricant give no clear idea of its usage. On the one hand, few studies report a positive influence of hBN in solid lubrication [3,10,74,75,76], while others state its negative or no effect on lubrication [77,78,79,80]. A decrease in CoF and wear rate with an increase in temperature up to 800 °C in Ni/hBN coating on stainless steel was perceived [75]. Conversely, a drop in friction and wear properties with an increase in sliding temperature in NiCr/hBN self-lubricating composite was reported in [80]. Wear reducing behavior of Ni60-10% hBN coating at 300 and 600 °C sliding was conveyed in ref. [79]. A similar content of hBN (10 wt.%) as inclusion was recommended in [80]. A reduction in HT CoF and wear rate was seen for Ni-based composite due to a synergism of Ag and hBN [81]. An extensive review points to 5–15 vol% of hBN as the optimal reinforcement in the composites to impart effective lubrication at a temperature range of 600–900 °C [81,82,83]. A larger hBN concentration resulted in significant deteriorating mechanical properties of materials. Figure 7 demonstrates the effect of sliding temperature on CoF and relative wear rates for various hBN based solid-lubricating materials available in recent literature [80,81,82,83,84,85,86]. The relative wear rate values are calculated after dividing the wear rate value at the reported temperature by room temperature (RT) value.

#### 2.2.5. Tungsten Disulfide (WS_2_)

WS_2_ is an excellent solid lubricant due to its laminar structure, where tungsten (W) atoms occupy the center layer of the hexagon, while the sulfur (S) atoms reside on the top and bottom layers of each hexagon (Figure 5a). The mechanism behind its friction reduction is due to the easy sliding between the adjacent layers, which are held by weak van der Waal forces similar to the structure of MoS_2_ [87]. Nevertheless, WS_2,_ unlike MoS_2,_ is conveyed to work at higher temperatures with their oxidation specified to be at 540 °C (for MoS_2_-350 °C) [88].

**Figure 8:**
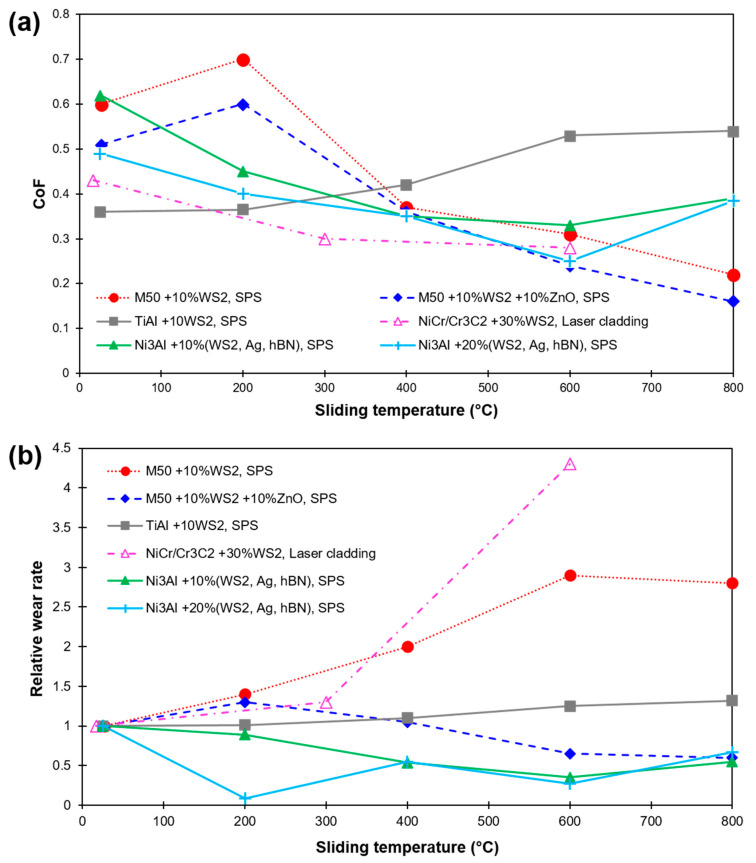
HT sliding results of WS_2_-based composites and coatings as found in recent literature (**a**) CoF; (**b**) Relative wear rate (in relation to their corresponding RT wear). Values are labeled as per WS_2_ concentration, matrix material, and fabrication method [10,87,88,90,91,92].

A significant reduction in friction and wear rate for Cu/WS_2_ composites due to the formation of the beneficial hard phase of W and lubricating phase of Cu_2_S was reported in [89,90]. The inclusion of WS_2_ in the Cu matrix demonstrated an improved mechanical and tribological property as compared to the composite with the same concentration of graphite due to the high strength of interfacial bonding between the Cu matrix and WS_2,_ resulting in a decreased plastic deformation (cracks formation) during sliding passes. Exceptional lubrication in the temperature range from 25 to 800 °C with WS_2_ and ZnO addition to TiAl matrix was demonstrated due to the formation of WS_2_ rich lubricating films (thickness ~500 nm) at temperatures below 600 °C and effective lubrication by ZnO beyond 600 °C [91]. A similar mechanism of wear was demonstrated in M50 steel+10%WS_2_ after wear tests under different temperatures [87]. At RT to 400 °C, the composite exhibits a delaminated worn surface. However, at high temperatures from 600 to 800 °C, the composite exhibits a mildly rough surface. In addition, at 600 and 800 °C, WS_2_ is damaged by oxidization and loses its lubricious effect to form wear debris of oxide particles (consist of WO_3_) which raises the friction coefficient [87]. In another study of 30%WS_2_ laser clad on Cr18Ni9 austenitic stainless, a decline in CoF and wear rate of the coating was demonstrated at all the testing temperatures (RT, 300 and 600 °C) [92]. However, the coating underwent decomposition and oxidization, resulting in no lubrication effect at 600 °C. Figure 8 exhibits the effect of sliding temperature on CoF and relative wear rate of several WS_2_-based composites and coatings as reported in recent literature [10,87,88,90,91,92]. The wear rates are relative to the material’s corresponding room temperature values (material’s wear rate at a particular HT dived by their RT value).

### 2.3. Alkaline-Earth Fluorides

Alkaline-earth fluorides such as LiF, CaF_2_, and BaF_2_ are well-known to provide solid lubrication at HT of 500–900 °C [3]. This is due to the reason that the material (CaF_2_) exists at a slip plane (compacting Ca atomic plane), and at HT conditions, the atomic force in the phase decreases, resulting in an easy shearing. However, alkaline-earth fluorides demonstrate poor tribological behavior at low-to-moderate temperature ranges. The responsible mechanism of friction and wear reduction in fluorides of alkaline-earth metals are reported to be their ‘softening’ around 500 °C, termed as the ‘transition point’ from a brittle to plastic or ductile state [93]. At low-to-moderate temperatures, they tend to be brittle, resulting in amplified wear (mainly abrasion) due to the third body effect [94]. Though the introduction of both mixtures of CaF_2_ and BaF_2_ to decrease the lubrication temperature to 400 °C as a result of lowering the melting point of composites is also reported in ref. [95]. Incorporation of rare earth trifluorides such as LaF_3_, NdF_3_ to reduce friction and wear at HT are also conveyed in ref. [96].

An improved friction and wear property of the SPSed ZrO_2_(Y_2_O_3_) matrix composites with an inclusion of 31 wt% BaF_2_ and 19 wt% CaF_2._was demonstrated at temperatures beyond 400 °C [97]. The CoF of the composite stabilized around 0.4, while it escalated for the reference material up to 1 at 800 °C. At RT sliding, the composites demonstrate poor behavior with signs of significant plastic deformation and delamination. A considerable decrease in friction and wear of Al_2_O_3_-50 wt% CaF_2_ composite at 400 °C was reported in [98], while a further decrease by two orders of magnitude at 650 °C, in comparison to the reference Al_2_O_3_ was noted. The formation of a Ca-rich lubricious layer on the surface of composites was held responsible for it. However, delamination of the formed lubricious tribolayer was seen at 800 °C, resulting in unstable friction. Similar to the previous works by ref. [96,97] the composites performed poorly at RT.

**Figure 9:**
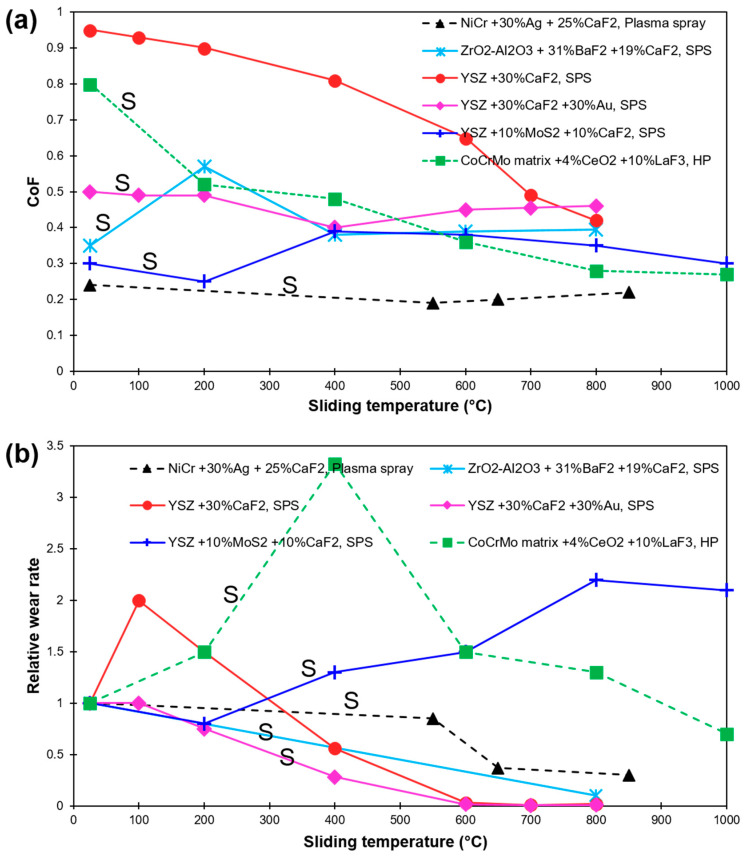
HT sliding results of Fluorides-based composites and coatings as found in recent literature (**a**) CoF; (**b**) Relative wear rate (in relation to their corresponding RT wear). Values are labeled as per Fluorides concentration, matrix material, and fabrication method [10,33,97,98,99]. Synergic effect of more than one solid lubricant is labeled with S.

Cura et al. reported a synergetic effect of Ag and CaF_2,_ resulting in enlarging the lubrication range from 200 to 650 °C [99]. Additionally, widening of lubrication range through the use of CaF_2_ and Au lubricants in the ZrO_2_(Y_2_O_3_) matrix was studied in [33]. At 400 °C and beyond, the composites demonstrated the formation of a smooth CaF_2_ lubricating layer, including Au lubricants. A similar effect of synergism was shown by others [100,101,102]. Figure 9 demonstrates the effect of sliding temperature on CoF and relative wear rate of several fluoride-based composites and coatings as reported in recent literature [10,33,97,98,99]. The wear rates are relative to the material’s corresponding room temperature values (material’s wear rate at a particular HT dived by their RT value).

**Figure 10:**
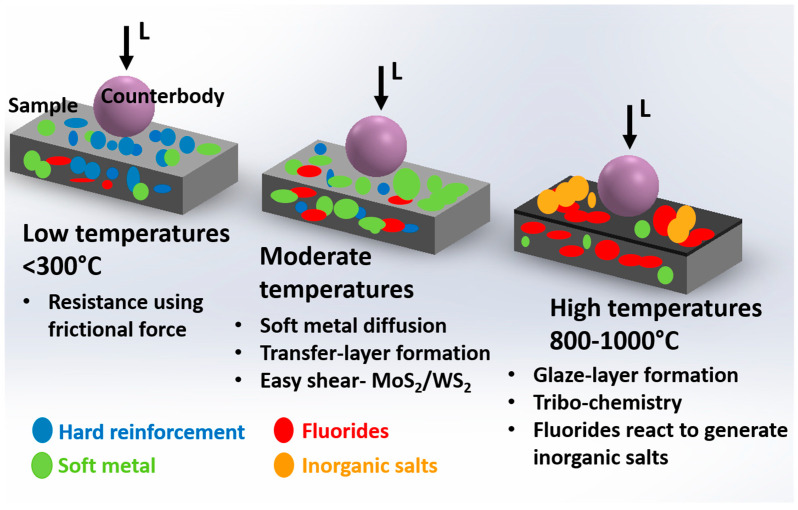
Schematic showing synergism of solid lubricants, i.e., soft metal/laminar solids and fluorides to broaden the range of lubricating temperature.

In general, the mechanism of synergic lubrication can be divided into three steps: (1) tribo-chemistry—at HT, the generation of lubricious compounds from the reaction between fluorides and matrix material occurs, resulting in low friction. (2) Transfer-layer—at moderate temperatures, the formation of tribo layer or transfer layer (rich in solid lubricants) protects the direct contact between tribo-bodies and thus minimizing friction and wear. (3) Glaze layer—at extreme HT, the formation of an oxide-rich glaze layer (with lubricating compounds) is seen. The glazed layer is reported to carry a wear-resistant and friction-reducing property [1,4]. A schematic of the synergetic effect at HT is presented in Figure 10.

### 2.4. Lubricious Oxides and Magneli Phases

In order to expand the range of working temperature of materials to around ~1000 °C or more, often oxides of metals are sought [3]. Lubrication from oxides is a complicated subject. Several works have been performed in this regard to report the HT lubrication range of various metal oxides in regards to their stability, adhesion strength, and lubrication mechanism. However, in situ formation of oxides is regarded as the most efficient method due to their abrasive nature at lower temperatures [103]. Generally, under a certain condition of temperature and chemical composition, a transformation (due to changes in the crystal chemistry of oxides) from brittle-to-ductile proceeds, resulting in their easy plastic deformation. Additionally, the tribological response of such oxides also depends on their respective ionic potential [10].

**Figure 11:**
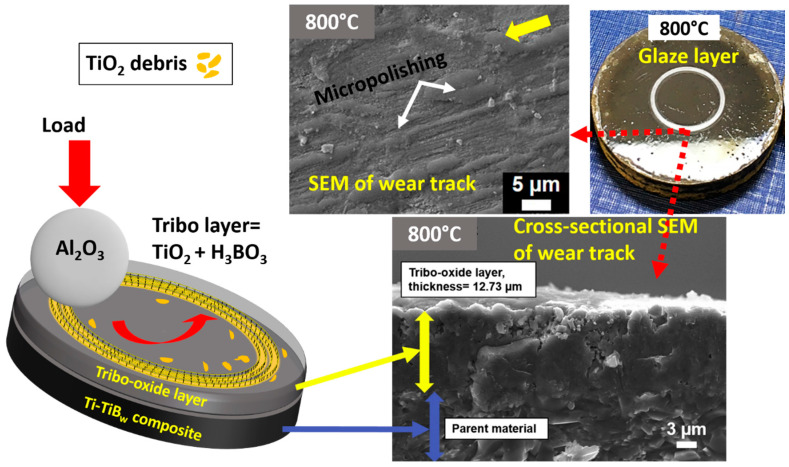
A collection of images from ref. [4] showing the mechanism of wear reduction at HT due to the formation of thick, homogeneous tribolayer. Tribolayer consisted of TiO_2_ and boric acid.

The protective behavior of oxides due to their optimum thickness, adherence to the substrate, a large load-bearing capacity, and a balanced Pilling–Bedworth ratio (PBR) during dry-sliding is reported in [104]. The developed oxide layer (or tribo-layer) at this point is thick enough to prevent contact between tribo-bodies and thus, resist wear. However, spalling, delamination and plastic deformation of the generated oxide layer is usually reported at low to moderate temperatures. A significant decrease in wear at 800 and 900 °C sliding due to the formation of thick, well-adhered, and hard rutile TiO_2_ phase on the surface of the Ti-TiB_w_ composite is demonstrated in ref [4] (Figure 11). Apart from the protective tribo-oxide layer, the formation of a glazed layer on the surface of composite, as well as counter body during 800 °C, sliding was also stated (Figure 11). The generated glassy-glazed layer at 700–900 °C on the surface of Ti-TiB_w_ carried a friction-reducing property (CoF~0.18). A similar formation of glassy layer phase of Cu_2_O was reported to diminish friction and wear of Cu-TZP composite at 600 and 700 °C (CoF-0.35 and 0.4 respectively) [105]. A similar lubricious effect of CuO (CoF~0.2) was reported by a few others [106,107]. The self-lubrication from lead monoxide (PbO) due to its soft, ductile and efficient shearing ability at HT is well-known. Nevertheless, their toxicity to the environment and human health has significantly limited their usage, putting them out of the main study.

**Figure 12:**
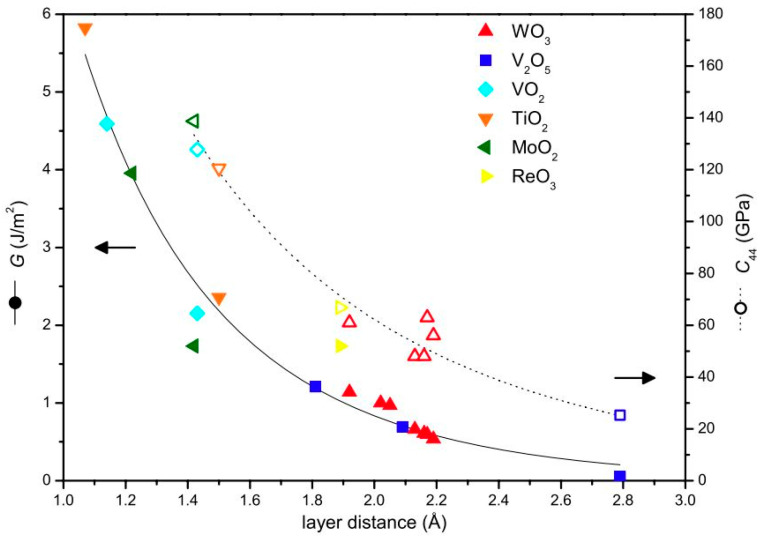
Contemplation of oxides forming Magnéli phase based on their de-cohesion energy (G) and elastic constant (C_44_) as a function of the distance between the cleaved layers [109].

For few oxides with a low melting point, the self-lubricity is demonstrated due to their quick melting; on the other hand, for others, the shear strength is relatively lower in specific crystal orientations owing to an absence plane of oxygen atoms; known as a crystallographic shear (CS) plane [108]. The latter has the tendency to impart low friction due to their lattice structure and are termed as Magnéli phase oxides. In addition, due to their high chemical stability, they are stated to show significantly low tribo-oxidation and adhesion to the counter body material. Figure 12 shows a relationship between de-cohesion energy (energy required to separate the cleaved layers) and elastic constant of certain oxides reported to demonstrate Magnéli phases [109]. A greater layer distance leads to lower de-cohesion energy and elastic shear constant, which is a degree of shear strength (Figure 12). As per calculation, V_2_O_5_ has the maximum layer distance and henceforth the lowest energy requisite to detach its layers, which specifies that its CS oxides will have superior lubricious nature than its peers.

The Magneli phase formation due to the oxidation of W-N coating during HT sliding is reported to impart a significant decrease in CoF and wear of the coating beyond 200 °C [110]. In another study [111], the lubricious Magnéli phase of Mo_4_O_11_ in Al_2_O_3_-Mo composites demonstrated the lowest CoF of 0.27 at 400 °C sliding due to their shear in the lattice of MoO_3_. Few studies on the W-O system [112,113] report the transformation of Magnéli phase oxide of WO_3_ to WO_2.9_ during sintering, further resulting in a considerably diminished CoF (up to 0.10).

Titanium shows a higher inclination towards oxygen and actively reacts to form oxides (TiO_2_) [114]. Few studies on titanium report Magnéli phases formation (transformation from TiO_2_) of gamma-Ti_3_O_5_, Ti_5_O_9_, Ti_9_O_17_ (Ti_n_O_2n−1_), and TiO_1.93–1.98_ [115,116] during HT sliding; resulting in a noticeable reduction in the shear strength on the surface as well as in the bulk materials. A drop in shear strength from 21 MPa to 8 MPa upon a transformation in stoichiometry from ~TiO_2_ to TiO_1.93–1.98_ was conveyed in ref. [116].

**Figure 13:**
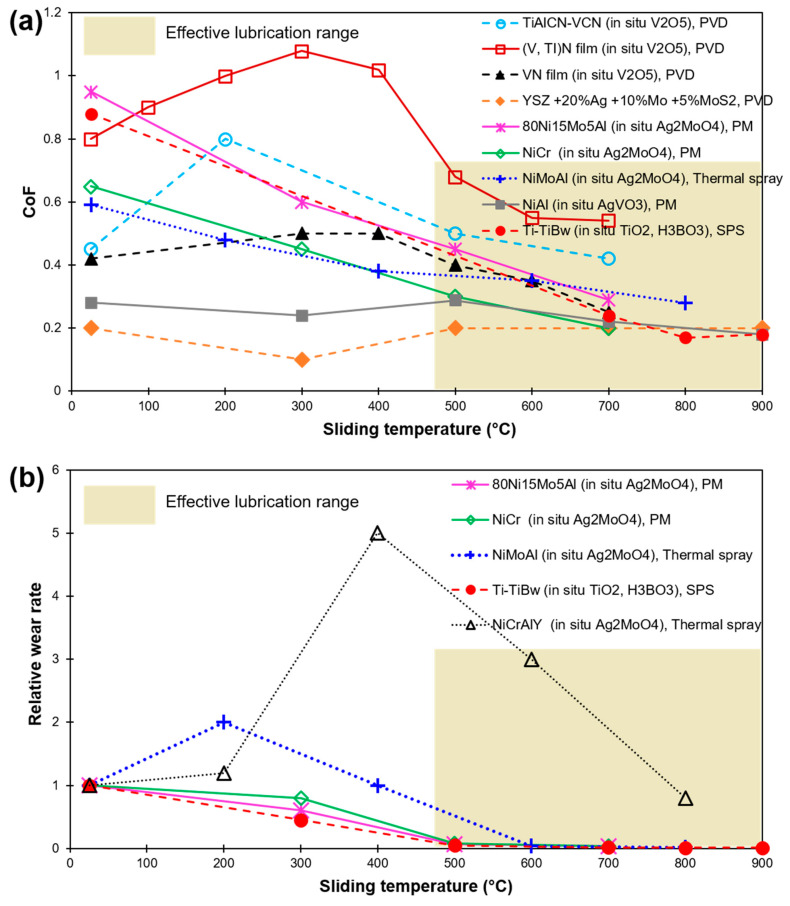
HT sliding results of oxide-based lubrication and coatings as found in recent literature (**a**) CoF; (**b**) Relative wear rate (in relation to their corresponding RT wear). Values are labelled as per oxide formation (in situ), matrix material, and fabrication method [4,13,118,120,123,124,125,126,127].

Vanadium oxides are stated to form similar Magnéli lubricious phases as that of titanium, analogous to the general formulations V_n_O_2n−1_ and V_n_O_2n+1_, with V_2_O_5_ [117]. The de-cohesion energy of the layers of V_2_O_5_ is the lowest resulting in an easy crystallographic shearing (Figure 12) [109]. The formation of wear-reducing phases of V_2_O_5_ and TiO_2_ on (V, Ti)N coating at 500–700 °C resulted in CoF to drift around 0.5 [118]. However, at 700 °C, a jump in CoF value to 0.6 was noted due to the coating damage and partial melting. Similar phenomena of coating melting close to 680 °C were stated in [119]. Particularly, Magnéli phases of vanadium show a steady decrease in CoF at temperatures between 400–700 °C due to liquid lubrication resulting in an easy-to-shear microstructure by V_2_O_5_, VO_2,_ and V_6_O_13_ [120,121,122], accounting for the melting and smearing of such phases. However, a complete melting and vaporization of V_2_O_5_ around 700 °C results in a lubrication failure [118,119,120,121,122,123]. Figure 13 demonstrates the effect of sliding temperature on CoF and relative wear rate values of composites and coatings forming oxides (and Magnéli phases), as reported in recent literature [4,13,118,120,123,124,125,126,127]. The efficient lubrication range is shaded.

### 2.5. Challenges, Opportunities, and Concluding Remarks

Production of solid-lubricating materials via powder sintering is widely reported. Limitations such as grain growth, poor mechanical properties due to a long holding time during sintering, etc., have surfaced to a quicker sintering technique, i.e., spark plasma sintering (SPS). However, a more efficient, faster, and low-energy consuming technique known as microwave sintering (MS) is equally heightening [128]. The quick heating incurred during MS due to the energy transformation rather than energy transfer (as in SPS) results in volumetric heating, further giving rise to a much finer and uniform microstructure. Apart from powder metallurgy (sintering), PVD techniques to fabricate solid-lubricating coatings have been widely observed. On the other hand, laser claddings to produce thick solid-lubricating coatings have been greatly undervalued despite their effectiveness. Deposition of a single layer using laser cladding technique is studied by several. However, very few exist on multilayer deposition due to limitations in mechanical property of sub-layer during re-melting [129].

The potential of additive manufacturing (3D printing) to fabricate bulks or coatings through layer-by-layer deposition is not yet reported. The possibility to generate complex geometries of solid-lubricating materials can be of high importance. Alternatively, the production of ‘smart’ solid-lubricating materials [3] demonstrating variations in their mechanical and chemical behavior upon an applied external stimuli using an approach of 4D printing is also foreseen [130].

**Figure 14:**
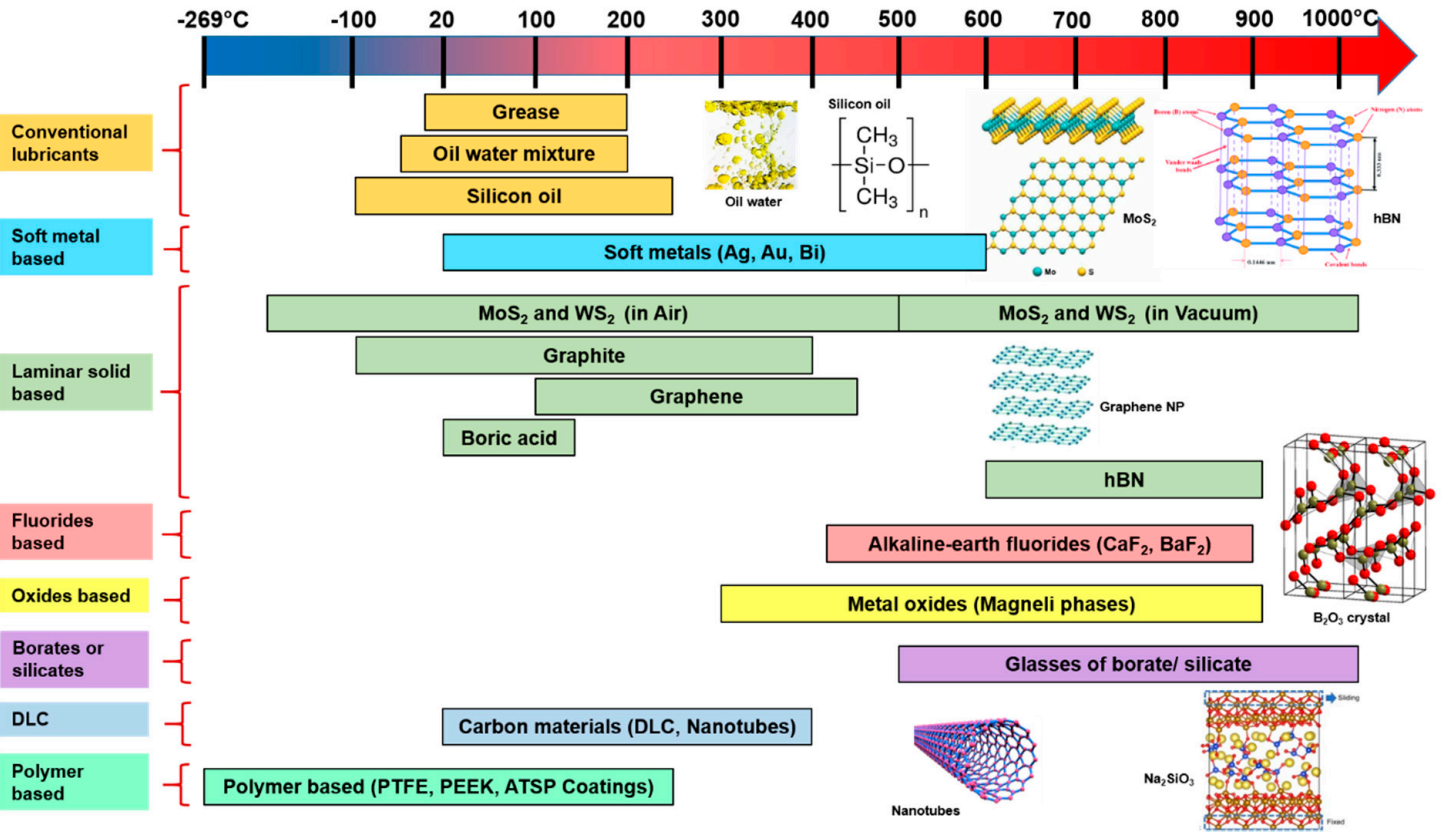
A graphical representation of effective temperature ranges for solid-lubricating materials (solid lubricants).

**Figure 15:**
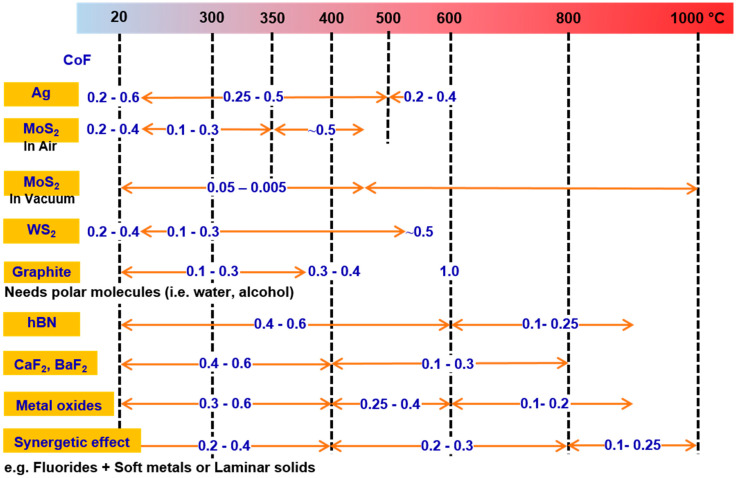
An approximate range of CoF under effective temperature ranges for widely used solid-lubricating materials (solid lubricants).

Figure 14 and Figure 15 demonstrate a graphical approximation of effective temperature range and their corresponding CoF for various groups of solid-lubricating materials (solid lubricants), respectively. In general, no single material exists that can cope with the complete tribological demands of working from room-to-extremely high temperatures (~1200 °C). However, the combination of various solid lubricants (such as soft metals, fluorides, etc.) to widen the lubrication temperature range (up to 1000 °C) is perceived (described in former sections). In order to accomplish the extreme temperature tribological needs, an HT solid-lubricating material should be designed as per the following considerations: (a) a CoF value below 0.2, (b) wear rate below10^−6^ mm^3^/Nm, and to work on a wide-ranging temperature from cryogenics to HT. There is a need for a more detailed study to understand the synergism of solid lubricants to provide lubrication under the aforementioned considerations over a wide temperature range.

The promising study of new age ‘diamond like carbon’ (DLC) coatings to minimize friction (up to <0.01) and wear is reported in few studies [131,132]. Their ability to regulate surface chemistry and structure under sliding conditions has conferred it to be ‘adaptive’ in nature. In addition to this, the combination of DLC with other solid lubricants such as TMDs, soft metals, etc., is stated to increase the lubrication range [133,134]. A low CoF and long endurance (operation) in dry/humid environmental sliding conditions under humid air, vacuum, and dry nitrogen atmosphere was reported for DLC-based nanocomposite coatings of WC/DLC/WS_2_ phases [133]. The CoF was 0.1 in humid air, 0.03 in a vacuum, and 0.007 in dry nitrogen. However, more research is needed for DLC-based coatings to understand the phenomena of surface adaptation and wear mechanism, especially during synergetic effects with other solid lubricants.

The current study brings into consideration the dependence of several factors during the self-lubrication of solid lubricants such as environment, operating conditions, the preciseness of testing methods, etc. Most of the work is based on a sliding test, which is incapable of a detailed comment on the behavior of solid lubricants in a dynamic mode of operations [135]. In addition, there exists a major lack of atomistic- and nano-level analysis of the evolving physical and chemical properties of surfaces and/or sub-surfaces, which is expected to broaden the understanding behind discussed mechanisms of operation. There still remains an unclarity in the details of feedstock/precursors composition and their methodical study, raising the concern for the correct experimental inputs. In this regard, the approach of simulation possibly will open the doors for better understanding about the effect of inclusions, their concentration and morphology, chemistry and evolution of buried sliding surface, predicting new inclusions, their reactions with the host matrix, the effect of the environment (cryogenics, vacuum), etc. on the lubrication behavior of solid-lubricating materials.

**Figure 16:**
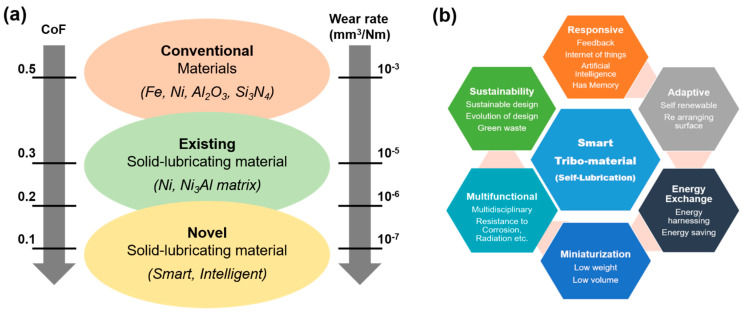
(**a**) Evolution of an HT solid-lubricating material in terms of CoF and wear rate; (**b**) features of a ‘smart’ solid-lubricating material [3].

With an increasing demand for materials to perform at extreme temperature applications to reduce friction and wear in the present industrial revolution, there is a parallel approach to save the environment, energy, and incurred life cycle cost. This exponential rise in material developments has not only propelled us towards environment-friendly footsteps but also towards designing a ‘smart’ tribo-material, which can be perceived as more efficient and multifunctional in approach (Figure 16). In addition to being adaptive and re-structurable, the new generation of tribo-materials is expected to show properties such as bio-mimicking (inspired from nature such as human skin, snake skin, fish scales, etc. to minimize friction, erosion) [16] and the ability to self-diagnose (such as, in fiber-reinforced plastics, useful in fast damage diagnosis, etc.).

Multidisciplinary studies in the designing of solid-lubricating materials are also foreseen. Cross-connection of tribology with other areas of physics, materials, mechanical engineering, and biomedical might help to strengthen the investigation in the design of a multifunctional and smart tribo-material. Clubbing with other areas such as information technology is believed to make advancements in ‘self-diagnosis’ and ‘repair’ through the use of artificial intelligence [3,136,137]. It is certain that under the canopy of a multidisciplinary approach, the tribology of HT solid/self-lubricating materials will take a leap from ‘self-adaptive’ to ‘smart’ to ‘intelligent’ lubricating material.

## Figures and Tables

**Table 1:** Key characteristics of high-temperature solid lubricants (self-lubricating material).

Characteristics of High-Temperature Solid-Lubricants
Demonstrate low shear strength. Adequately high cohesion strength of lubricious film formed at HT so as the film does not break upon high load and/or friction. Mechanical strength, thermal and chemical stability, oxidation and corrosion resistance. High thermal conductivity in order to dissipate heat. Controlled depletion during tribological operations.
